# Amplification of Asynchronous Inhibition-Mediated Synchronization by Feedback in Recurrent Networks

**DOI:** 10.1371/journal.pcbi.1000679

**Published:** 2010-02-19

**Authors:** Sashi Marella, Bard Ermentrout

**Affiliations:** 1 Center for Neuroscience/Center for Neural Basis of Cognition, University of Pittsburgh, Pittsburgh, Pennsylvania, United States of America; 2 Department of Mathematics, University of Pittsburgh, Pittsburgh, Pennsylvania, United States of America; École Normale Superieure, College de France, CNRS, France

## Abstract

Synchronization of 30–80 Hz oscillatory activity of the principle neurons in the olfactory bulb (mitral cells) is believed to be important for odor discrimination. Previous theoretical studies of these fast rhythms in other brain areas have proposed that principle neuron synchrony can be mediated by short-latency, rapidly decaying inhibition. This phasic inhibition provides a narrow time window for the principle neurons to fire, thus promoting synchrony. However, in the olfactory bulb, the inhibitory granule cells produce long lasting, small amplitude, asynchronous and aperiodic inhibitory input and thus the narrow time window that is required to synchronize spiking does not exist. Instead, it has been suggested that correlated output of the granule cells could serve to synchronize uncoupled mitral cells through a mechanism called “stochastic synchronization”, wherein the synchronization arises through correlation of inputs to two neural oscillators. Almost all work on synchrony due to correlations presumes that the correlation is imposed and fixed. Building on theory and experiments that we and others have developed, we show that increased synchrony in the mitral cells could produce an increase in granule cell activity for those granule cells that share a synchronous group of mitral cells. Common granule cell input increases the input correlation to the mitral cells and hence their synchrony by providing a positive feedback loop in correlation. Thus we demonstrate the emergence and temporal evolution of input correlation in recurrent networks with feedback. We explore several theoretical models of this idea, ranging from spiking models to an analytically tractable model.

## Author Summary

Neurons in many parts of the brain fire spikes rhythmically and synchronously in many behaviorally and functionally relevant contexts. There are many mechanisms for producing oscillatory synchronization between populations of biological oscillators. One way to produce synchrony is that the population of oscillators receives common correlated input. In this paper, we study a population of oscillating neurons (mitral cells) that are not directly coupled to each other but receive broadband correlated input from a second population of neurons (granule cells). The granule cell population, in turn, receives inputs from the mitral cells; hence, the mitral and granule cells are reciprocally connected. Correlated input to the oscillating mitral cells produces tighter synchrony in the activity of the mitral cell population. We hypothesize that this increased mitral cell synchrony will evoke greater activity in specific groups of granule cells and that these specific granule cells, in turn, become the source of the correlated input to the mitral cells. That is, the synchronous input from the mitral cells increases the fraction of correlated feedback. Thus, we close the correlation loop. We show through analysis and simulations that this feedback mechanism can lead to the spontaneous appearance of highly synchronous activity within the mitral cells. We show that there is good experimental support for this mechanism in the circuitry of the olfactory bulb. We speculate that such mechanisms could also arise in other parts of the brain.

## Introduction

Synchronization of neural activity has been suggested to facilitate coding [1]–[3] and propagation of activity [4]–[6]. Synchronous stimulus-induced oscillatory activity has long been known to occur in the olfactory system of mammals [7]–[11]. Synchronous, rhythmic activity has been proposed to play a role in odor discrimination tasks [12]. In insects, disruption of synchronous oscillations can impair discrimination of chemically similar odorants [13]. In mice, enhancement of synchronous oscillations in the olfactory bulb using genetic modifications improves performance in fine discrimination tasks [14]. In the mammalian olfactory system, mitral cell synchrony contributes to the generation of the gamma oscillations in the local field potential; for example, in the cat olfactory system, increases in the synchrony between mitral cells are accompanied by a concomitant increase in the power of the gamma band in the local field potential [15]. Mitral cells have been shown to undergo synchronization during odor-evoked responses [16] or during olfactory nerve stimulation [17]. Although, previous experimental and modeling studies have highlighted the role of granule cells [18] and lateral inhibition [19] in the production of gamma oscillations in the olfactory bulb, the exact mechanism by which such mitral cell synchronization occurs in the mitral-granule cell network connected by reciprocal recurrent and lateral connections remains largely unknown.

A possible mechanism of synchronization of mitral cells in the olfactory bulb is suggested by recent experimental evidence. In paired recordings from mitral cells, activation of a mitral cell elicits fast unitary inhibitory post-synaptic potentials (IPSC's) in a second mitral cell [17],[20],[21]. These IPSC's are due to the synaptic activation of the shared granule cells via the mitral-granule cell dendrodendritic synapses. Although the individual IPSC's are fast, they arrive randomly (asynchronously), i.e. the output of the granule cells is not time locked to the stimulus. The temporally prolonged barrage of these unitary IPSC's produced in response to the spiking in the first mitral cell results in a slow rising and long lasting hyperpolarization in the second mitral cell [20]–[23]. There is a variable delay between the evoked IPSC's in the second mitral cell and the spike times in the first mitral cell [20]. Thus, the evoked IPSC's occur asynchronously [20]–[22], aperiodically [21] and the kinetics of hyperpolarization in an ensemble average of the evoked IPSC's show a slow rise time (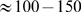 ms) and a slow decay constant (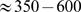 ms) [20]–[22]. In addition, the peak amplitudes of the ensemble average are small, (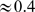 mV) [20]. The prolonged, asynchronous barrages of IPSC's have been shown to be a result of long latency, asynchronous and long lasting mitral cell recruitment of granule cells [23]. Furthermore, recent experimental studies into the origin of synchrony between mitral cells suggests that recovery from shared IPSC inputs from common granule cells is the primary driving mechanism for mitral cell synchrony [17],[21]. These physiologically measured properties of mitral-granule cell interactions suggest a novel mechanism of synchronization of mitral cells in the olfactory bulb.

Previous studies have proposed that noise can synchronize oscillators [24]. For neurons to undergo such noise-induced synchronization they should be periodically firing and should have some shared fast fluctuations in their inputs. Recent studies on the mechanism of generation of synchronized oscillatory activity by long lasting asynchronous, aperiodic inhibition in the olfactory bulb have revealed exactly such a novel role for noise [21]. It was shown that two mitral cells firing in the gamma frequency range can undergo synchronization upon receiving common inhibitory input from granule cells. The degree of synchronization was shown to depend on the degree of correlation in the noisy input shared by the two neurons. Although spiking was synchronized, the shared noise itself was aperiodic. In all of the experimental and theoretical studies of stochastic synchronization to date, the degree of correlation is imposed and held fixed. In our study the degree of input correlation emerges intrinsically from within the network and is amplified over time due to the dynamics of the network. In addition, our study utilizes theoretically derived probability distribution of phase difference for uncoupled oscillators receiving shared noise to investigate the conditions necessary for the existence of bistability in the magnitude of input correlation. Here we consider the case in which correlated fluctuations from granule cells arise naturally from granule cells that connect to many mitral cells. The input correlation to any pair of mitral cells could increase if the shared pool of presynaptic granule cells increased their stochastic firing rate thus providing a greater amount of common noise. In the olfactory bulb, synapses between mitral and granule cells are dendrodendritic, and almost always reciprocal [25]. Thus, if a granule cell synapses on a pair of mitral cells, those mitral cells also synapse on that granule cell. We hypothesize that, since a pair of mitral cells with correlated input is more likely to fire synchronously, this pair is also more likely to provide correlated input to their common granule cell. In turn the common granule cell could then increases its release of transmitter increasing the correlation to the mitral cells. The result of this is that the feedback provides an amplification of correlation. The goal of this paper is to use computational and analytic techniques to show that such feedback will increase correlation and as a consequence, synchrony between oscillating mitral cells.

We describe three models for feedback induced correlation and stochastic sychronization. We first study one pair of mitral cells and one common granule cell. The mitral cells are modeled as simple phase oscillators which are perturbed through their phase-resetting curves (PRCs). The granule cell is modeled as a noisy leaky integrate and fire (LIF) neuron receiving synaptic input from the mitral cell oscillators. The second model replaces each phase oscillator with the conductance-based Morris-Lecar oscillator. Finally, to allow for analytic approaches, we reduce the first two models to a discrete time map which we study using an averaging technique.

## Results

### “Spiking” Models

**Figure 1:**
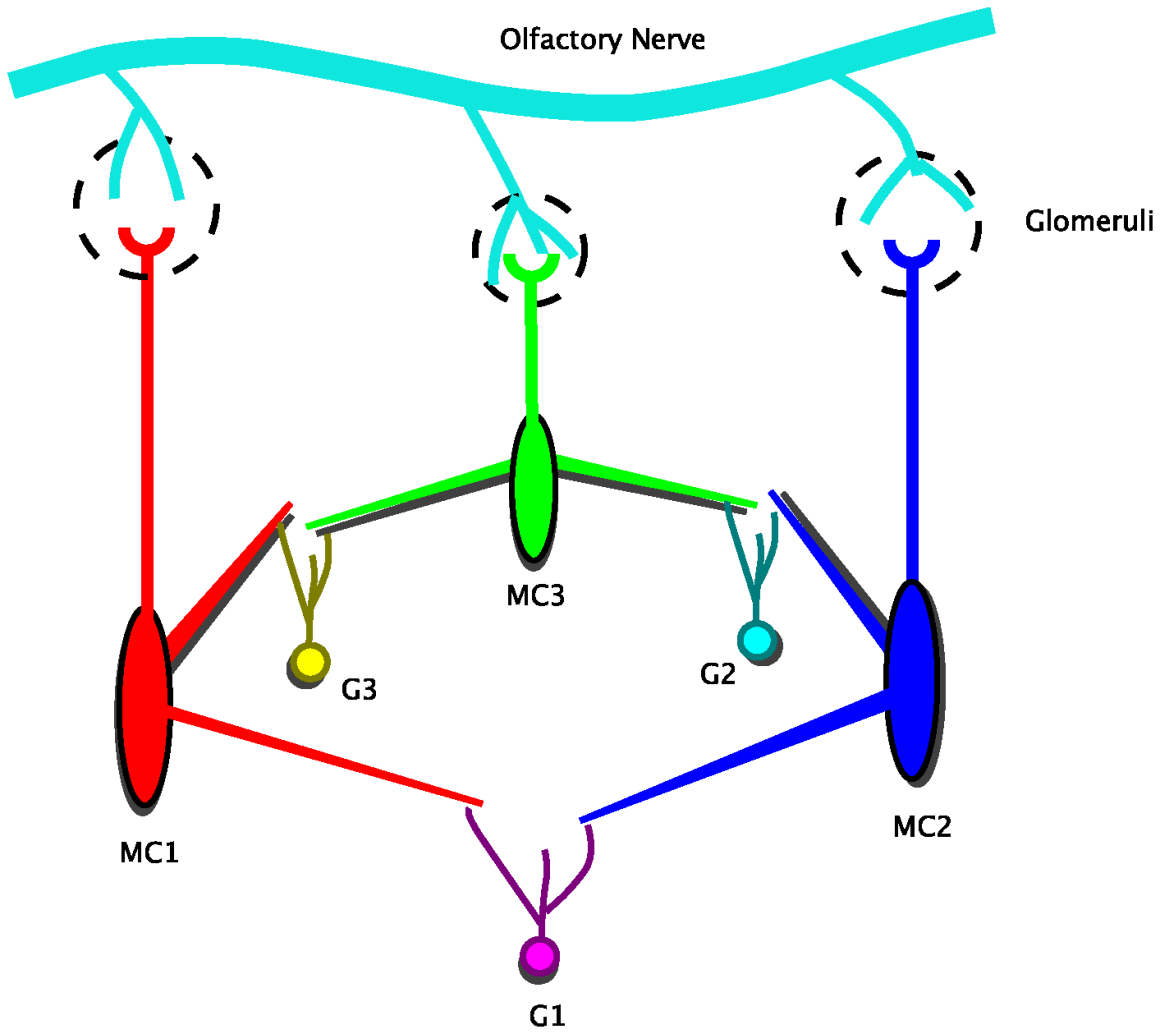
Schematic depicting the network architecture. The generalized (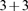) network with 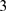 mitral cells and 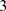 granule cells is shown here. The simplified (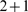) network lacks mitral cells (MC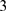).

During odor inputs or stimulation, mitral cells fire in a narrow frequency range, so that we can regard them as limit cycle oscillators [26]. Any oscillator can be represented by a single phase variable [27], so we first consider a such pair of mitral cells as phase oscillators:
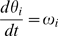
where 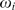 is the natural frequency of the oscillator. These oscillators receive input from a shared granule cell which is modeled here as a noisy leaky integrate-and fire (LIF) neuron:
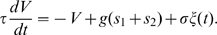


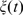
 is a white-noise process and 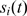 are the synaptic inputs from the two mitral cells:
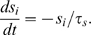
Each time 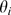 crosses 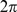, the synaptic input, 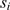 is incremented by 1. To model the long-lasting synaptic bombardment by the granule cell, we introduce a variable, 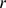 which satisfies
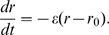
Each time the granule cell fires (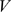 crosses its threshold, here set to 1), 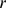 is incremented by 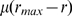 and 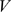 is reset to 0. 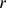 represents the rate of the shared Poisson process. This shared Poisson process represents the feedback via dendrodendritic synapses from the population of granule cells to the mitral cells, the rate of which is dependent on the spiking activity of the granule cells. In addition, there are two independent Poisson processes (independent sources of noise) with fixed rates, 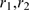. Each of these three Poisson processes generates events which we regard as the brief random inhibitory post synaptic potentials seen in patch clamped mitral cells [17]. We suppose that the effect of these inputs on the mitral cell oscillator is to shift the timing of the next mitral cell spike by an amount that depends on its current phase. The function that determines this shift is called the *phase resetting curve*, denoted, 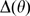 which has been computed for many types of neural oscillators, including mitral cells [28]. If oscillator 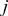 receives an input, then its subsequent phase (and thus timing) is given by, 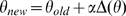, where 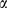 is the magnitude of the kick. If the input is generated by the shared processwith rate 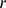, both 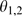 are incremented while if the event is generated by the process with rate 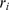, only oscillator 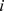 is incremented. In our simulaftions, we choose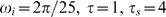,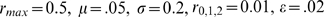, 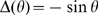 and 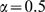. We vary the coupling, 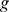 to the granule cells from the mitral cells between 0 and 2. We will refer to the above network construction (2 mitral cells and 1 granule cell) as the 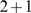 network. We also made a network consisting of three oscillators (mitral cells) and three granule cells (LIF). Oscillators 1,2 drove LIF 1, 1,3 drove LIF 2, and 2,3 drove LIF 3. Oscillator 1 received Poisson input from LIF 1,2; 2 from 1,3; and 3 from 2,3. All other parameters are the same. This network will be referred to as the 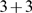 network as depicted in Figure 1.

**Figure 2:**
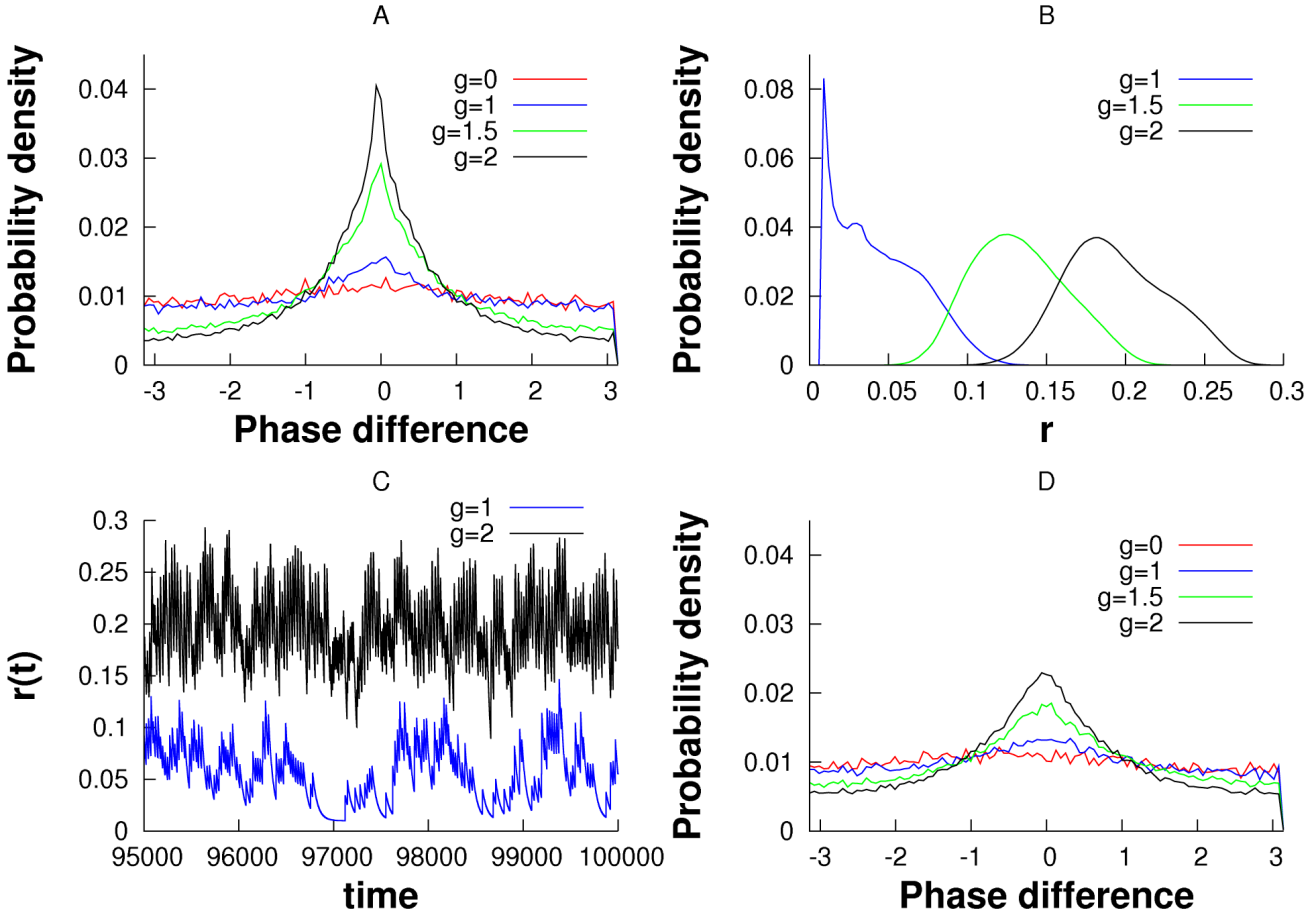
Self-organized synchronization in a stochastic feedback network of two mitral cells and one granule cell(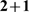 network). (A) Probability density of the phase-difference 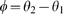 for different strengths of input to the granule cell. The peak at zero phase difference increases with strength of the synapse. (B) Distribution of the values of 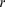, the shared Poisson rate of the granule cell for different strengths of the synapse. (C) Plots of 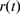 for 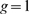 and 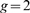. (D) Phase difference histograms for the 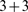 network. The central peak exists without decay for even larger network sizes (data not shown) suggesting that stochastic synchronization is robust against larger network sizes.

There are several ways to quantify synchrony in oscillator networks. For phase models in which the phase is explicit, it is convenient to look at the histogram of the phase-differences, 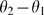; the more peaked is this histogram, the closer to perfect synchrony (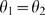) are the two oscillators. Figure 2 depicts simulations of the 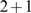 network. Figure 2A shows a histogram of the phase-differences, 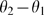 as a function of the coupling from the mitral cells to the granule cells. When the LIF granule cell is uncoupled from the mitral cells, 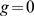, the histogram is nearly flat as the rate of shared input is the same as the unshared input and both are quite low. There is a small peak due to the small degree of correlation. As the coupling to the granule cell increases, the peak of the histogram becomes much sharper since the firing of the granule cell is now dependent on the spiking of the mitral cells. As a consequence of this sharpening, the rate of the shared input, 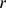 increases as shown in the histograms of Figure 2B for identical values of 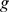. It is important to understand that the firing rate of the shared granule cell population, 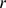 indicates the input correlation in the mitral cells that share these granule cells, which in turn represents the magnitude of synchronization of these mitral cell activities. Hence we use 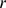 as a stand-in for synchronization in the mitral cells. The probability distribution of 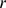 can also depict the stability of the input correlation (and hence synchronization) in the system. If the distribution is bimodal it indicates the existence of bistability in the input correlation in the mitral cells (and their synchronization). Thus in Figure 2B, 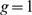 is interesting since it appears to be slightly bimodal,i.e. the distribution of 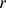 has two peaks for 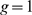. Figure 2C shows a segment of the temporal dynamics of 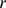 for 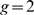 and 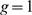. Figure 2D shows a simulation of the 3 mitral and 3 granule cell network. The peaks are not as sharp as in Figure 2A for similar input strengths. This is because oscillator 1 gets two strong inputs from granule cell 1 and granule cell 2 and thus shares correlations with the *two* other mitral cells putting a limit on the maximum correlation from a single cell. We also simulated a larger network (with 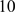 mitral cells and 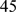 granule cells) to confirm that the central peak in the probability distribution of the phase differences does not decay with increase in network size (results not shown).

**Figure 3:**
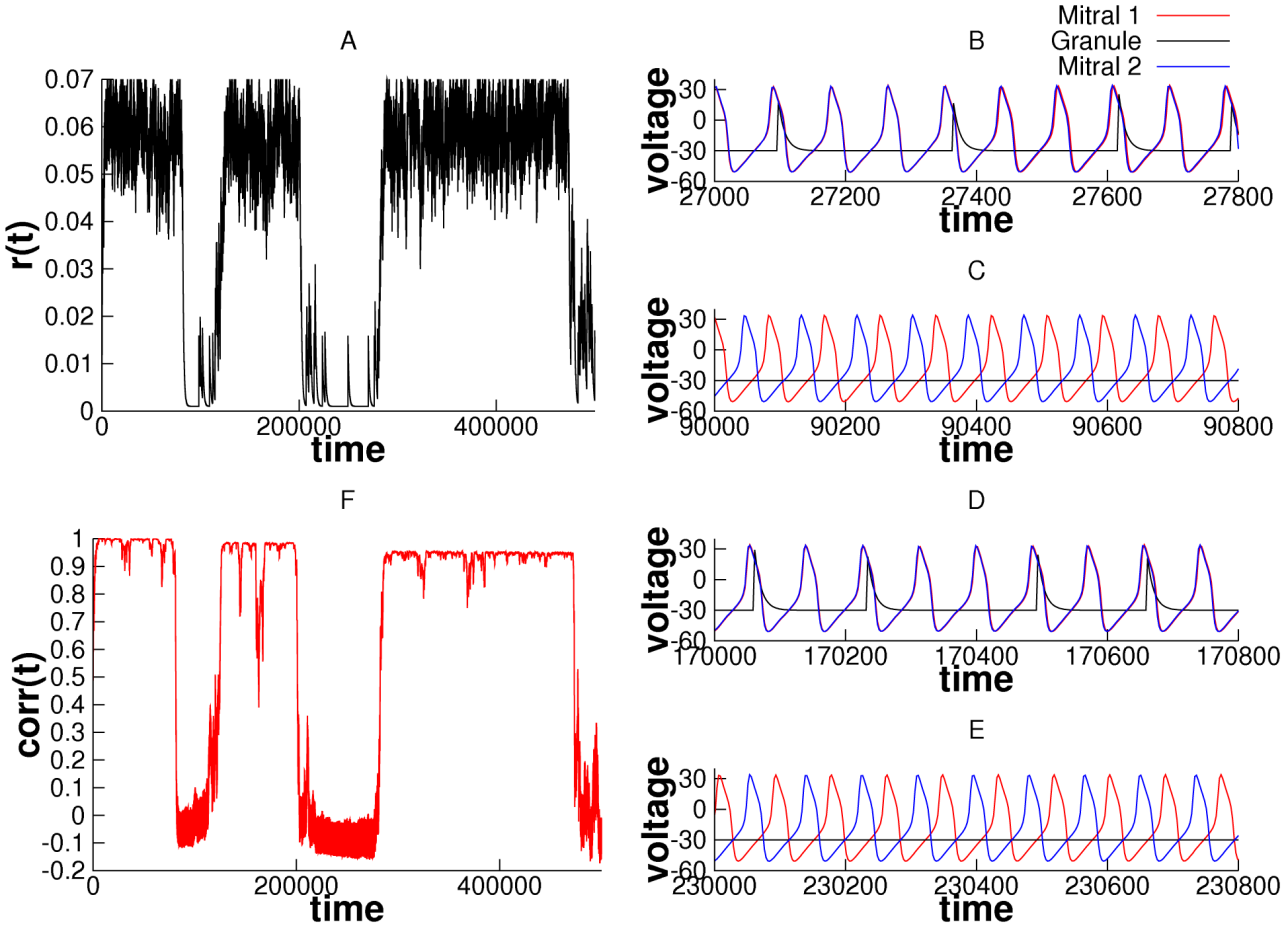
Self-induced stochastic synchrony between a pair of Morris-Lecar model neurons and a leaky integrate-and-fire model neuron. (A) Rate of release of the LIF, “granule cell” showing switches between synchrony and asynchrony. (B–E) Sample voltages at four different time points corresponding to time in A, showing synchrony when 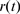 is high and asynchrony when 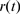 is low. High(low) granule cell activity during synchronized(unsynchronized) mitral cell activity can be observed. (F) Correlation coefficient calculated for the voltage data between the two mitral cells.

In Figure 2, we modeled the “mitral” cells as a pair of simple phase models. There is similar behavior when we replace the phase oscillators with conductance-based models such as the Morris-Lecar model but with very pronounced bistability. Figure 3 shows a sample simulation with the same set up as in Figure 2, but the phase models are replaced by the Morris-Lecar oscillator. Since phase is difficult to obtain, we instead look at the correlation between the voltages over a moving time window (see methods). There appears to be two “attractors”; one where the oscillators are completely uncorrelated and 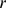 is low and the other when they are tightly correlated and 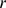 is high. This is suggestive of the possibility of bistability. Figure 2C (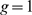) shows a similar bistability between the synchronized and desynchronized state. We suspect that intrinsic noise in the system effects the switch from one to the other and the positive feedback maintains the states for a long period of time.

**Figure 4:**
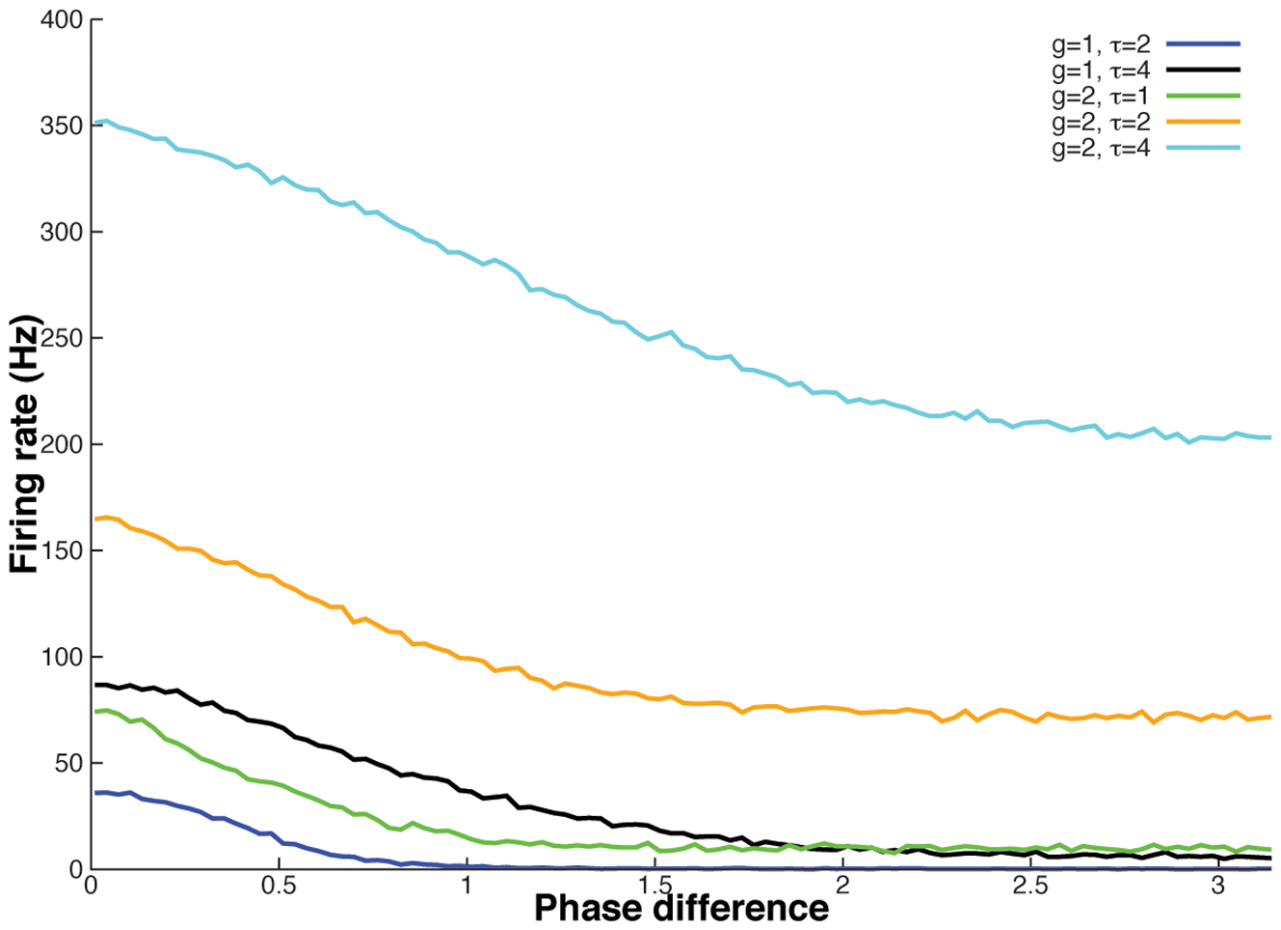
Dependence of the total spike count of the granule cell on the phase-difference of the two oscillators for different input strengths (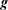) and integration times of the synapse (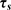). Higher dependence of the firing rate on the phase difference is observed for weaker and shorter synapses. The firing rate is less dependent on the phase difference for stronger and longer synapses.

We can begin to understand the mechanism of amplification of synchronization by considering the dynamics of 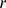. We suppose that 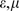 are small so that we can average 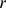 and see that its value depends on the firing rate, 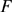 of the LIF:
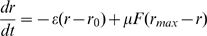
(1)
Figure 4 shows how the LIF firing rate, 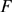, depends on the phase difference between the two oscillators, 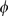. Here we count the number of spikes in a time window of 10 seconds to determine 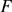. The shape of this function depends on 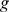, the time constant of the synapse, 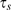 (as well as other parameters such as 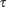 and 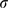). In general, this is a decreasing function of 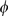. As the strength of the synapse, 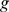 increases or as the decay of the synapse, 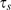 increases, the spike count is larger and depends less on the phase-difference between the oscillators. For small 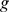 and short-lasting synapses, the LIF is a coincidence detector and depends very strongly on the timing difference of the inputs. Thus, for 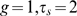, if the phase difference between the two oscillators is more than about 0.75 radians (corresponding to about 3 msec for oscillators running at 40 Hz) then there will be almost no firing of the LIF. Similarly, for 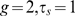, (green), the timing difference should be less than 6 msec. For larger 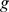 and longer synapses, the LIF always fires and the ratio of the minimum to the maximum rates is only modestly small.

We can now see the basic principles underlying the amplification of stochastic synchronization. Initially, 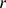 is low and the shared granule cell fires at a very low frequency. The phase difference between the two oscillators drifts, and thus, on occasion the two mitral cells fire nearly synchronously. This increases 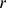 transiently and thus increases the correlation of the inputs to the oscillators. This in turn increases the rate at which the shared granule cell fires, further increasing 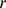 resulting in a positive feedback loop and finally mitral cell synchronization. In the next section we derive a more abstract model which we are able to analyze.

### Reduced Model

We start with exactly the same model as above for the mitral cells: a pair of phase oscillators. However, instead of explicitly modeling the LIF and its synaptic excitation we consider only the 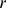 equation (1) which will be incremented according to the degree of synchronization of the two mitral cells. That is, we replace 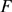 by an explicit functional of the phase-difference between the two oscillators. As above 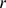 sets the rate of a Poisson process that produces events which excite *both* mitral cell oscillators. Similarly, there are two independent processes with fixed rates 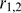 which provide background unshared noise to the two mitral cell oscillators. Let 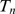 be the time interval between events for these three Poisson processes. We choose 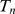 from an exponential distribution with rate 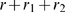 and then choose which of the three events has occurred according to the relative sizes of 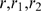 (as per the Gillespie algorithm [29]). We can then reduce the behavior of the randomly perturbed oscillators to a map and thus use the theory developed in [30] to determine the density of the phase-differences. Specifically, let 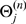 denote the phase of oscillator 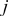 after the 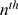 kick from a population of granule cells (common and independent projections). Then
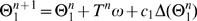
(2)

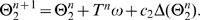
(3)

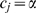
 if oscillator 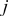 is kicked and is zero otherwise. Thus, if the event was generated by the common process with rate 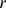, 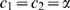, while if it was generated by the independent process, say, 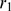 then 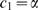 and 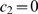. These equations simply say that the phase of each oscillator at the 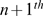 granule cell spike is equal to the phase at 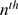 granule cell spike advanced by the phase traversed by the oscillator given its angular frequency 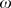 in the 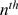 inter-spike interval 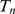. If the oscillator receives the 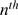 granule cell spike (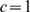), an additional phase advance/delay dictated by the phase resetting curve, 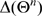 is added to obtain the actual phase of the oscillator at the 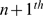 granule cell spike, 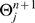. The probability of both oscillators receiving granule cell input simultaneously (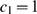,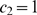) is 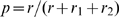. The probability of either one of the oscillators receiving granule cell input (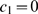, 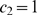) and (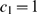, 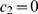) is thus 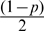. To simulate this process, we generate two random variables, one to determine the interval between inputs, 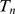 drawn from an exponential distribution and the other drawn from a uniform distribution to determine which of the three pairs, 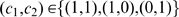 occurs.

In a previous study [30], 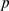 was assumed to be constant. Here, since 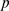 is proportional to the rate of the common granule cell which is, in turn, proportional to the degree of synchronization of the mitral cells, we allow 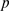 to evolve on a slow scale similar to equation (1):
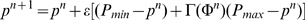
(4)The functional 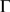 could depend on the instantaneous phase-difference between the mitral cell oscillators 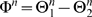 or some time averaged version of it. We discuss several choices in the next section. However, we assume that 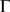 gets larger when the two oscillators are more synchronous (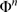 near zero) and small when they are not synchronous. Thus, when 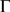 is large (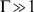), 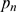 will slowly evolve toward 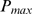 while when 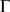 is small (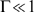), it will decay toward 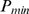. In terms of the original models with the LIF, 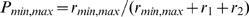.

### Choice of 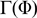


There are at least two plausible ways to choose 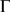 a direct and indirect way. In the direct way, we assume that 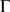 is a function of 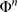, while in the indirect version, 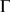 is a function of some time averaged version of the phase-difference, such as an order parameter. We will discuss the direct choice first.

#### Single stable fixed point

**Figure 5:**
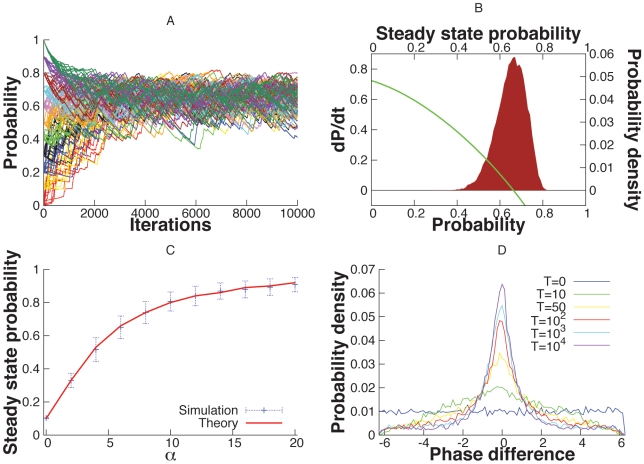
Evolution of 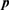 in the presence of a single stable fixed point. (A) The temporal evolution of 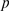 from various initial states. All initial states are attracted by the single stable fixed point. (B) Histogram of the final values of 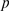 in different trials from (A). The green curve depicts the numerically calculated values of equation 7 (C) The dependence of the median probability on the amplitude of 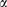. (D) The curves depict distribution of phase differences drawn at various time points from simulations such as in (A). A slow development of synchrony on the order of hundreds of milliseconds is observed.

Figure 4 shows how the firing rate of the “granule cell” depends on the phase-difference 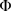. The probability of shared input is proportional to this rate, so a natural choice for 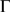 is proportional to the firing rate 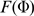 depicted in the figure, for example, 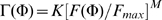. If 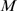 is large, this creates a highly peaked function of the phase difference with a maximum at zero. We use the following approximation of such a function:
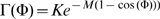



With this choice for 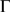, equations (2, 3, 4) constitute a simplified discrete dynamical system to represent the models from Figures 2 and 3. Figure 5A shows the evolution of 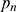 over time with 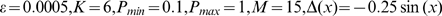. After a long transient, the stochastic variable, 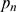 tends to a fairly sharp density function centered around 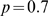 (see Figure 5B). At the same time, the phase-difference, 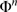 evolves on a fast scale to a highly peaked distribution centered at 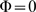 as shown in Figure 5D. Here, we let the oscillators evolve according to equations (2, 3) for 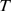 iterations. We see that in the early stages, the density of phase difference is flat but becomes peaked as the simulation evolves in time. We can vary the magnitude of the function 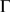, 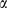 and examine the steady-state value of 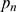. This is shown in Figure 5C. In order to analyze this equation, we exploit the assumption that 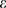 is small. Since 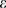 is small, we can apply averaging and approximate the dynamics of 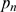 by the dynamics of the averaged equation, 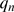 satisfying:
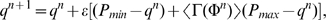
(5)where 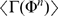 is the average value of 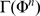. In order to calculate 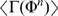, we require 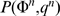, which is the probability density of phase difference 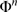 given 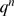. Since 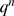 evolves slowly, we can treat it as constant allow the oscillators to evolve until they reach a stationary density. In [30], we obtain an analytic formula for the steady state density, 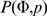, the density of phase-differences, 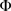 given a probability, 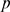 of common input. From this, we obtain:
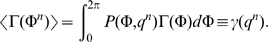
(6)


Hence, we can analyze this case by finding the stable fixed points for the averaged dynamics:
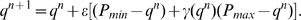



The fixed points satisfy:
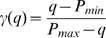


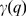
 is typically a bounded non-negative increasing function of 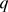. The right-hand side less than or equal to zero at 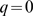 and has a vertical asymptote at 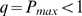, so that there is always at least one stable fixed point between 0 and 1. For our simple choice of 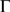 there is exactly one stable fixed point for 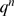. In Fig 5A, the model was allowed to evolve from random, uniformly distributed initial phase difference between the two oscillators and various uniformly distributed initial values for 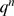. It is seen that irrespective of the initial conditions, the system evolves towards a single stable fixed point for 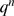. The theoretically predicted value of the stable fixed point agrees well with the median of the distribution of the steady state 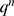 values from many trials, as seen in Figure 5B. The green curve shows the function,
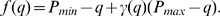
(7)


The position of the stable fixed point for 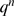 depends on the magnitude of 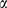. At small values of 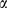, the steady state lies close to 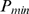. For larger values of 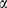, the steady state 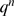 increases monotonically towards 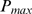. The predicted steady state values match well with Monte-Carlo simulations as can be seen in Figure 5C. In Figure 5D, it can be seen that the evolution of synchrony evolves over time over a time scale of 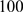 ms, as can be observed from the distribution of phase difference at different points during the simulation.

Before moving to the next section, we can ask whether or not there is more than one stable fixed point to the averaged dynamics. We conjecture that there will not be. The reason for this is that in [30], we show that the probability density, 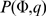 has the form:
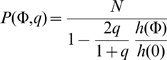
where 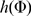 depends on the shape of the PRC, 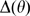. Integrating 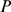 against 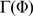 yields a function of 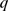 which for small 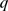 depends linearly on 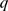 and saturates to 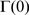 as 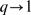 (since 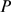 approaches a delta function). Thus, 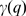 is roughly like
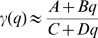
with 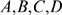 positive no matter how we choose 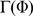. For this approximation, it is easy to show that 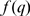 has at most one positive root. Thus, we expect no bistability between a synchronous and an asynchronous state. In order to get bistability, there should be an inflection point 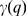, for example by having 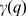 depend sublinearly on 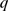 for 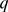 small, e.g., 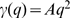 for small 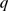. We will study a choice of 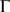 that produces bistability in the next section.

#### Bistability

When 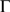 is an *instantaneous* function of 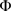, then there appears to be no bistability between asynchrony and synchrony. To produce a model which exhibits the kind of bistability shown in the full model (e.g. Figure 3A,B), we will assume that 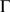 is a function of some temporal average of the phase difference. That is, instead of averaging over a nonlinear function of the phase, we apply a nonlinear function *after* performing some averaging. Before discussing how such a rule could be biologically implemented, we consider a simple choice for this rule. A common measure of synchrony [27],[30] is the circular variance (or “order parameter”):
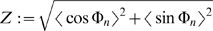



We can write this order parameter as a function of the of the density, 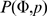:
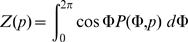
since 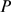 is an even function of 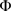 and in our previous work [30], we showed that 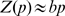 for 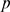 small; that is, it is linear. The results in the previous section show we need nonlinear dependence on 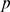, so we take
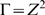
which will give us 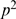 dependence for 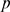 small.

**Figure 6:**
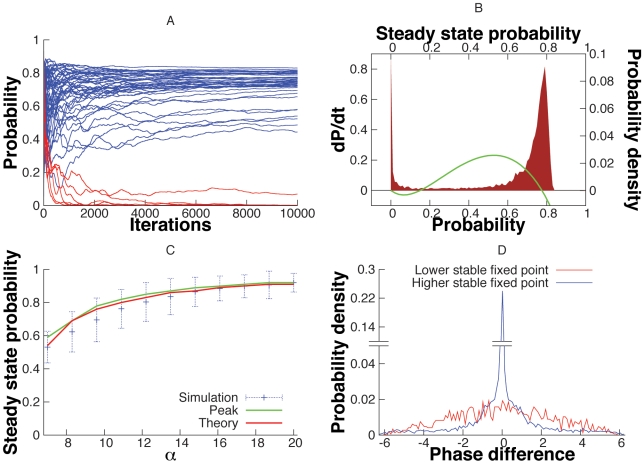
Evolution of 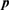 in the bistable regime. (A) The temporal evolution of 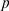 from various initial states. The initial states move randomly to either one of the stable fixed points. (B) Histogram of the final values of 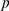 in different trials from (A). The green curve depicts the numerically calculated values of equation 7 for the indirect choice of 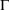. (C) The dependence of the steady state probability on the amplitude of 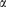. The taller peak of the bimodal distribution is depicted by the green curve. (D) Probability distribution of the phase difference between mitral cells for the two fixed points.

This choice of 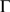 produces a fundamental change in the system's dynamics. The system with, 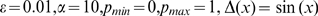, now displays two distinct stable states as seen in Fig 6(a), where the system with random uniformly distributed initial phase difference and 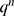 evolves either to a zero or a non-zero steady state 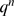. The steady state distribution of 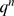 values reveals the two stable fixed points as seen in Fig 6b, both of which are predicted accurately by theory. The position of the non-zero stable state depends on the choice of 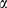. Fig 6(c) shows the agreement between the theoretically predicted value, the mean of the distribution of all final states higher than the unstable fixed point and the position of the peak in the distribution of the non-zero steady states. Finally, the two stable states differ in their synchronization. The zero stable state is characterized by oscillators with low synchrony whereas the non-zero stable state has oscillators with significantly higher synchrony as can be seen in Fig 6(d).

## Discussion

We have described a new mechanism for the amplification of oscillatory synchrony through feedback. Unlike previous models that depend on phasic oscillatory inhibition [31], our feedback is long-lasting (nearly tonic) and highly stochastic. Specifically, we study stochastic synchronization in a generalized network of mitral cells by inhibitory granule cell inputs which themselves receive dendrodendritic mitral cell feedback. The mitral cells are not directly (monosynaptically) coupled but are coupled disynaptically via the shared granule cells. Thus, the granule cells provide both the recurrent and lateral connectivity, as has been described in the mammalian olfactory bulb. We use spiking models with LIF neurons to demonstrate the feasibility of stochastic synchronization in the olfactory bulb with feedback from granule cells. We then use abstract models to analyze the mechanism of the self-organization as a result of the feedback-induced stochastic synchronization. Our models are based on experimentally observed kinetics of the mitral-granule cell interaction's. The key assumptions of our model, borne out in experimental studies are that the granule cell output consists of asynchronous, aperiodic, prolonged barrages of IPSC's with small average amplitudes and long ensemble decay constants. Such mitral-granule cell interactions have been observed experimentally using extracellular stimulation in the glomerular layers as well as intracellular stimulation of mitral cells [17], [20]–[23].

Fast synchronized inhibition has been shown to play a central role in producing synchronization in a sparse, randomly connected network of excitatory and inhibitory cells where the PING (pyramidal interneuronal network gamma) mechanism is observed [31]. However, in the olfactory bulb, mitral cells receive inhibitory postsynaptic potential (IPSC's) from granule cells in the form of asynchronous barrages with small average amplitudes [20]. In addition, the decay time constant of the probability envelope of these IPSC's is too long [17],[20],[21], for a PING-like mechanism to produce synchrony [32]. PING is based on fast inhibitory feedback which produces a “window of opportunity” for the excitatory cells to fire and thus requires strong inhibition. The synchrony induced by stochastic synchrony is not locked to the inhibitory events, but instead relies on the correlations in the “noisy” granule cell inputs shared by mitral cells. Here, we study the role of feedback in this system. Specifically, we propose that more synchronous mitral cell activity could produce activity of shared granule cells which would result in higher correlations in the input to the mitral cells. In other words, we propose a positive feedback loop in which the stochastic synchronization of mitral cells is enhanced by the correlated inhibitory output from granule cells, which in turn is enhanced by the correlated mitral cell spiking. As the synchrony is dependent on correlation of input from shared granule cells rather than fast transient inhibition, it is a distinct and separate mechanism from PING.

Olfactory bulb circuitry is unique in the central nervous system. The principle output cells, mitral cells, make synapses with the inhibitory granule cells through their dendrites rather than their axons. Activity of the granule cells produces long lasting recurrent and lateral inhibition which has two components: a long lasting slow component and a fast random component. The slow component acts to keep the spike frequency of the mitral cells in a limited range i.e. the firing rate of the mitral cells does not vary much with odor concentration [26], thus the slow asynchronous inhibition acts to balance the excitatory drive to the mitral cells. The fast component serves as “correlated noise” to synchronize mitral cell oscillations. Granule cells do not need to spike to produce inhibition , thus, with weak stimulation, effects of inhibition remain local and provide little correlation between mitral cells. However, if several mitral cells fire together, then this may be enough to cause the shared granule cell to fire spikes resulting in the widespread calcium release into granule cell dendrites and thus, all the mitral cells that are connected to that particular granule cell will receive fast correlated random inhibitory input [17] which results in lateral inhibition.

In the spiking model, we use a slow variable 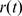 to describe a shared Poisson process whose rate is modulated by the spiking of the single common granule cell in the 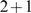 model. This process is used to mimic a population of common granule cells whose firing rates are modulated by synchronized firing of the mitral cells. This simplification is used in order to obtain a probability envelope of an ensemble average of shared granule cell inputs where individual granule cells are assumed to be Poisson processes. We show in the spiking model that stochastic synchronization can indeed be induced by the feedback loop between the mitral and granule cells.

We show that in the abstract model using general oscillators that a feedback loop between mitral and granule cell input can indeed synchronize mitral cell activity which is otherwise uncorrelated. The abstract model also provides important insight into the nature of dependence of the evolution of 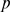 on the phase difference, 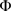 between the oscillators. Dependence of 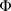 on a centrally peaked 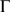, produces a system with only one stable steady state. On the other hand, if 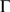 is an order parameter, then bistability between synchrony and asynchrony is possible in some parameter regimes. The spiking network also displays similar dependence on granule cell activity. Both the abstract and spiking models show a gradual temporal evolution of synchrony which is similar to observed evolution of synchrony in the olfactory bulb(see figure 2A in [17]). The Morris-Lecar model suggests the existence of bistability, even though the granule cell rate is dependent on the *instantaneous* (as opposed to time averaged) timing difference between the two mitral cells. This could be a consequence of the fact that the synapses to the mitral cell oscillators have temporal dynamics rather than being instantaneous. Interestingly experimental observations of desynchronized to synchronized shifts of mitral cell activity and vice versa [17](see figure 2C) seem to suggest the possibility of bistability in the input correlation (and synchrony of mitral cells) in the olfactory bulb. We conjecture that in the olfactory system, the mitral-granule network is monostable. But, if bistability should indeed exist, it would most probably be mediated by a slow process that accumulates coincident activity of mitral cells over time. Bistability might be common place in other cortical networks where such memory forming slow cellular processes might have evolved. In such networks, a transient increase in correlated inputs can push the system from one state to another, hence allowing for a transient correlation-induced dynamic switching behavior. Evoked IPSC's in a lateral mitral cell are known to occur with a variable latency [20]. Granule cell activity is also known to develop with a variable long-latency [23]. In addition, synchrony between mitral cells is known to develop with a variable delay (50–150ms) [17]. These latencies are thought to be a function of the stereotypical fashion in which mitral cells recruit granule cell activity and in turn experience a shaping of their own activities. Our models did not include detailed cell type specific morphologies but could reproduce the time dependent evolution of synchrony between mitral cells (see Figure 5D).

Both the simple and generalized network models have a notable dependence of their synchronization on the rate of decay of 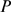. Finally, it can be seen that the phase difference of two mitral cells is highly dependent on the firing rate of the granule cell. These results taken together suggest that stochastic synchronization does play a major part in determining the activity of a network of mitral and granule cells in a closed loop with feedback connectivity. Although this investigation focuses on specific details of the olfactory circuit, the proposed mechanism is generally applicable to cortical circuits that include a subpopulation of neurons that provide long lasting, small amplitude, asynchronous outputs. We conjecture that the general mechanism proposed here could be at play in other parts of the brain where such asynchronous release with long decay times have been previously reported, for example, the hippocampal cholecystokinin-expressing interneuron-granule cell circuit [33]. In this study, we did not investigate the importance of synaptic depression and facilitation, or spike frequency adaptation in determining the absolute amount of synchrony or in mediating the switch from the asynchronous to synchronous states. Such a control of the amount of synchrony and/or switches between the two extreme states may also be mediated by top-down modulation of granule cell firing, hence allowing for a cortical control of mitral cell synchronization.

## Methods

### Morris Lecar Model

We used the general Morris-Lecar model with the following equations.
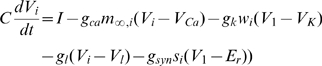


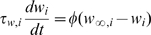


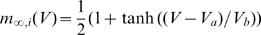


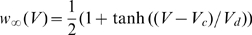


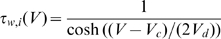


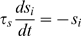
withparameters,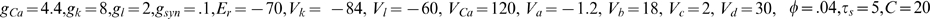.

To compute the correlations shown in Figure 3, we compute
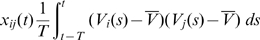
and plot 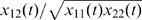.
